# A Comparative Study of Cocaine-Related Deaths Using Anti-Cocaine Antibodies as a Diagnostic Tool to Provide Spatial Information on Drug Distribution and Pathological Myocardial Responses

**DOI:** 10.3390/ijms27020698

**Published:** 2026-01-09

**Authors:** Paola Santoro, Donato Morena, Pierluigi Crusco, Alessandro Santurro, Matteo Scopetti, Vittorio Fineschi

**Affiliations:** 1 Department of Anatomical, Histological, Forensic and Orthopedic Sciences, Sapienza University of Rome, 00161 Rome, Italy; paola.santoro@uniroma1.it (P.S.); donato.morena@uniroma1.it (D.M.); pierluigi.crusco@uniroma1.it (P.C.); 2 Department of Medicine, Surgery and Dentistry “Schola Medica Salernitana”, University of Salerno, 84081 Baronissi, Italy; asanturro@unisa.it; 3 Department of Medical Surgical Sciences and Translational Medicine, Sapienza University of Rome, 00189 Rome, Italy; matteo.scopetti@uniroma1.it

**Keywords:** cocaine-related death, forensic pathology, immunohistochemistry, anti-cocaine antibody, cardiotoxicity, contraction band necrosis, postmortem diagnosis, oxidative stress, myocardial injury, toxicology

## Abstract

Cocaine-related deaths present significant diagnostic challenges due to the nonspecific nature of cardiac histopathology and the limited reliability of postmortem toxicology, often affected by redistribution phenomena. This study investigated the postmortem heart expression and distribution of an anti-cocaine monoclonal antibody, aiming to evaluate immunohistochemistry (IHC) as a potential complementary tool for diagnosing cocaine-related fatalities. Fifteen cases of acute cocaine-related death, with toxicological data exclusively positive for cocaine, were examined and compared to ten cases negative for drug abuse. Cardiac samples from the lateral left ventricular wall and interventricular septum underwent IHC using an experimentally optimized protocol. All cocaine-related cases demonstrated clear and widespread immunopositivity, with varying staining intensities across a semi-quantitative scale. Immunostaining localized consistently to nuclear and myofibrillar compartments and showed no association with postmortem interval (mean PMI 72.33 h; range 30–144). Control samples exhibited no staining. Positive immunostaining also highlighted cardiomyocyte alterations related to cocaine toxicity, particularly hypercontracted fibers with myofibrillar rhexis and contraction band necrosis. While these findings align with the established cocaine-induced myocardial injury, the intense nuclear staining observed may further reflect oxidative DNA damage associated with cocaine exposure. This study provides novel evidence supporting the applicability of anti-cocaine IHC in postmortem investigations. The technique may serve as a valuable adjunct in detecting cocaine distribution within cardiac tissue, particularly when toxicological data are inconclusive or unavailable.

## 1. Introduction

Cocaine abuse remains a major global health concern, with mortality rates showing a progressive rise over the past decades. An estimated 25 million people used cocaine in 2023, representing 0.47% of the global population between 15 and 64 years old. In the same age group, the prevalence was estimated at 0.24% among women and 0.70% among men [1]. In 2023, cocaine accounted for nearly 30,000 deaths in the United States alone, emphasizing its persistent public health burden [2].

Cocaine is a tropane alkaloid derived from the leaves of Erythroxylon coca, and is encountered in two forms: cocaine hydrochloride, a crystalline salt typically consumed intranasally, orally, or intravenously, and the free base (“crack”), which is volatilized and inhaled [3].

From a pharmacokinetic perspective, cocaine distributes rapidly throughout the body, exhibiting a volume of distribution of around 1–3 L/kg and binding to plasma proteins (albumin and α1-acid glycoprotein) at a rate close to 90%. High tissue concentrations are reached in the brain, spleen, kidneys, lungs, heart, and skeletal muscle [4]. Its elimination half-life ranges from 40 to 90 min. Metabolism occurs predominantly via plasma butyrylcholinesterase to ecgonine methyl ester (EME) and through tissue esterases to benzoylecgonine (BEG) [5,6]. Only a small proportion undergoes hepatic N-demethylation to norcocaine. Cocaine and its metabolites are eliminated primarily in the urine [7,8].

The forensic interpretation of cocaine concentrations in postmortem cases remains a major challenge. Measured drug concentrations after death do not necessarily reflect antemortem levels due to postmortem redistribution (PMR), which refers to the movement of drugs from tissues into blood and other body fluids after death.

Pathologically, cocaine abuse has been linked to a wide spectrum of organ damage. Reported lesions include cardiomyopathy, pulmonary injury (“crack lung”), dissecting aneurysms, pulmonary vascular remodeling with secondary hypertension, renal injury [9], and multi-organ infarctions [10].

In the cardiovascular system, catecholamine accumulation secondary to sympathetic overstimulation and inhibition of norepinephrine reuptake is thought to underpin the high incidence of arrhythmias, myocardial infarction, and sudden cardiac death observed in cocaine users [11]. The macroscopic and microscopic manifestations of cocaine on the heart, as well as its neurotoxicity, are derived from a variety of mechanisms, including mitochondrial dysfunction, oxidative stress, neuroinflammation, excitotoxicity, and autophagy. Some effects are determined by overstimulation of the adrenergic system. Cocaine causes coronary artery vasoconstriction, atherosclerotic phenomena, and thrombus formation. In this way, cocaine favors myocardial infarction. Chronic cocaine use has been demonstrated to result in concentric left ventricular hypertrophy, as well as progressive myocardial fibrosis. Furthermore, a correlation has been established between the use of the substance and a number of cardiac conditions, including myocarditis, dilated cardiomyopathy, and heart failure. [12,13,14,15,16].

Although these histopathological patterns are frequently encountered, none are pathognomonic for cocaine use, limiting the diagnostic specificity of routine histology in postmortem investigations. Consequently, toxicological testing remains the gold standard for confirming cocaine-related deaths.

This limitation highlights the need for complementary diagnostic approaches. Immunohistochemistry (IHC), a technique that combines morphological analysis with molecular specificity, has proven invaluable in both diagnostic and forensic pathology. By applying antibodies directed against cocaine or its metabolites, IHC has the potential to detect drug-related antigens within tissue sections, thereby providing spatial information on drug distribution and organ-specific pathological responses. Recent studies have suggested that such approaches may be particularly useful in postmortem settings where toxicological specimens are unavailable or degraded [17].

The present research aims to explore the application of IHC in the investigation of cocaine-related deaths. Specifically, this study aims to evaluate the relationship between cocaine concentrations in conventional matrices (e.g., blood) and immunohistochemical detection using an anti-cocaine antibody in the heart. The key goal is to assess whether IHC can serve as a reliable complementary or alternative diagnostic tool in forensic pathology, improving the capacity to establish drug-related death in cases where toxicological data are incomplete or unavailable.

## 2. Results

This study aimed to evaluate the correlation between cocaine administration and the cardiac distributive response in cocaine-related deaths, investigating the relationship between conventional toxicological findings and immunohistochemical features of the heart using an anti-cocaine antibody, with the objective of assessing its potential value as a new or complementary diagnostic marker.

Samples obtained from 15 cases of acute cocaine-related death, with negative toxicological analysis for other drugs, were analyzed through immunohistochemistry to evaluate the pattern of cocaine distribution in the heart.

**Figure 1:**
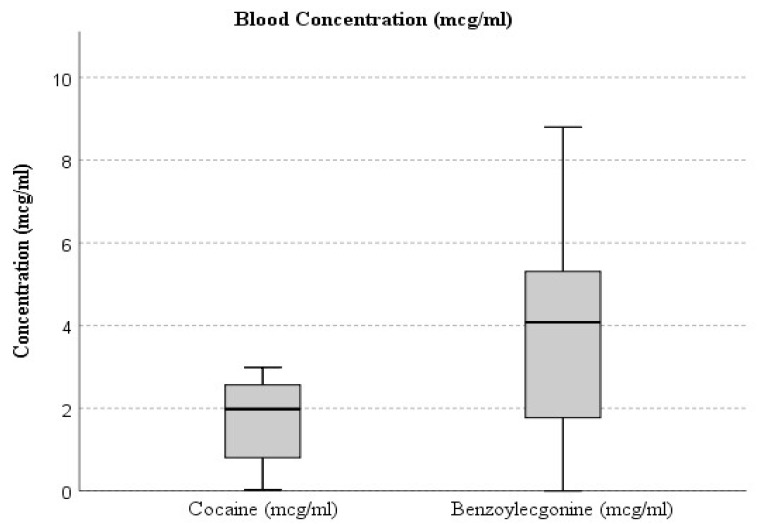
The box plot illustrates the distribution of cocaine and benzoylecgonine concentrations in blood samples.

The box plot in Figure 1 shows blood concentrations (mcg/mL) of cocaine and BEG in the study population. Cocaine shows a narrower range and lower median concentration, indicating limited variability and rapid clearance from the bloodstream. In contrast, BEG displays a broader distribution with higher maximum values, reflecting its slower elimination and greater stability as a primary metabolite of cocaine. The interquartile range (IQR) and whisker extension highlight the higher dispersion of BEG concentrations compared to cocaine, suggesting more prolonged systemic presence and accumulation over time.

The immunohistochemical technique allowed us to quantify the expression of the response to the anti-cocaine antibody. The reaction was classified as follows:

- 0 (−) not expressed;
- 1 (+) isolated and disseminated expression;
- 2 (++) expression in scattered foci;
- 3 (+++) expression in widespread foci;
- 4 (++++) widespread expression.

The table below (Table 1) shows the toxicological analysis of each case (cocaine and BEG concentrations in peripheral blood, urine, and bile) and summarizes the semi-quantitative evaluation of the immunohistochemical findings, also providing the gradation of the immunohistochemical reaction in heart samples (lateral left ventricular wall—LLVW and interventricular septum—IS).

The heart samples used as a negative control (from car accident deaths with no clinical history of drug abuse and negative toxicological analysis) showed no reaction in heart or blood vessels when stained with the anti-cocaine antibody.

Immunostaining was evident in all heart samples examined from the fifteen cocaine-related deaths. The mean semi-quantitative score was 2.93 (moderate to strong expression) in the lateral left ventricular wall and 2.87 (moderate to strong expression) in the interventricular septum. In lateral left ventricular wall samples, nuclear and myofibrillar staining was predominant and distributed in widespread foci. In contrast, a mainly nuclear staining with isolated to scattered foci was observed in the interventricular septum samples. The table below provides a detailed representation of obtained immunohistochemical semi-quantitative scores, sorted by each cardiac area examined (LLVW and IS) and by staining localization (myofibrillar and nuclear) (Table 2).

**Figure 2:**
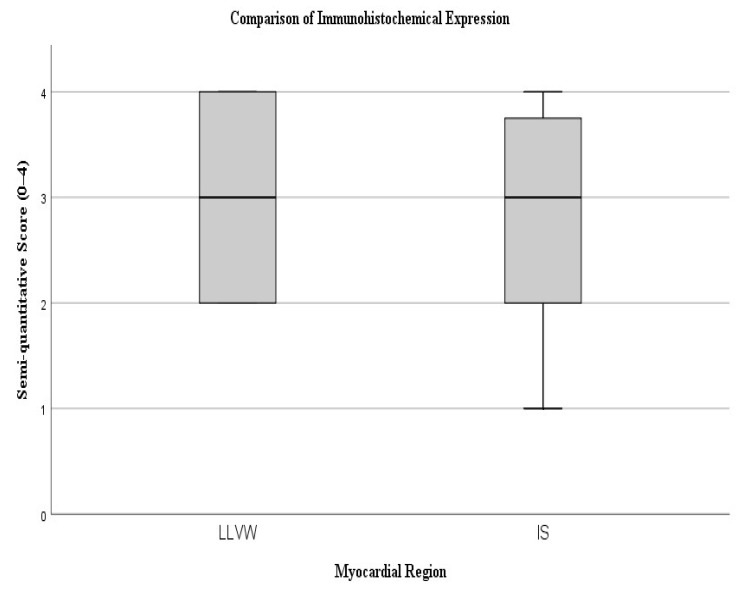
The box plot illustrates the distribution of semi-quantitative scores between the lateral left ventricular wall (LLVW) and the interventricular septum (IS).

A non-parametric Mann–Whitney U test was performed to evaluate whether the difference between the semi-quantitative scores was statistically significant. The analysis yielded a U value of 109.0 (*p* = 0.880), indicating that the difference between groups was not statistically significant. These findings suggest comparable levels of immunohistochemical expression between the two myocardial regions, with a trend toward slightly greater staining intensity in the lateral ventricular wall (Figure 2).

**Figure 3:**
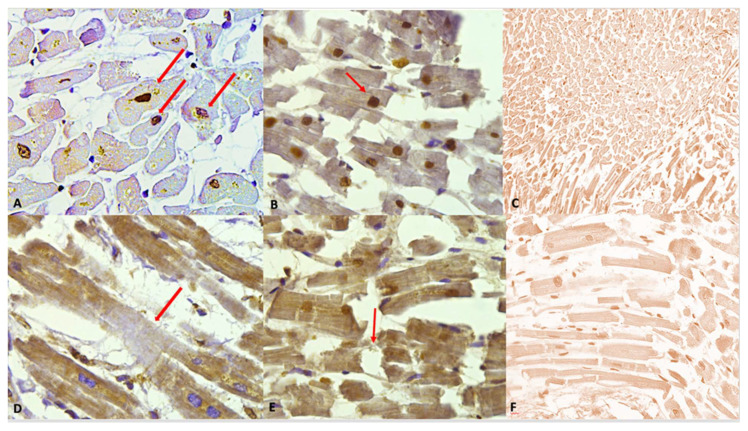
(**A**) Grade 1 (+): isolated and disseminated nuclear expression, demonstrated (red arrows) by nuclear brownish staining. 20×. (**B**) Grade 2 (++): expression in scattered foci. The nuclear brown reaction is more evident and intense. A slight reaction can be observed in myofibers, too. A nucleus with squaring of its edges is shown (red arrow) as a sign of cardiomyocyte damage. (**C**) Negative control: Any immunohistochemical reaction is shown. (**D**) Grade 3 (+++). Myofibrillar expression in widespread foci. The immunostaining is demonstrated by a clear brown reaction involving myofibers, compared to the negativity in the nuclei. An area of hyporeactivity in a degenerated myofiber is shown (red arrow). (**E**) Grade 4 (++++). Myofibrillar and nuclear widespread expression. The brown reaction is evident. Myofibrillar breakage is shown (red arrow). (**F**) Grade 0 (−). Negative control, magnification 40×.

The following figure (Figure 3) illustrates the immunohistochemical expression of the anti-cocaine antibody and its various grades of expression (according to a semi-quantitative scale), along with the different alterations observed in cardiomyocytes.

The observation of heart samples from cocaine-related deaths revealed a wide range of cardiomyocyte alterations, highlighted by the positive staining.

**Figure 4:**
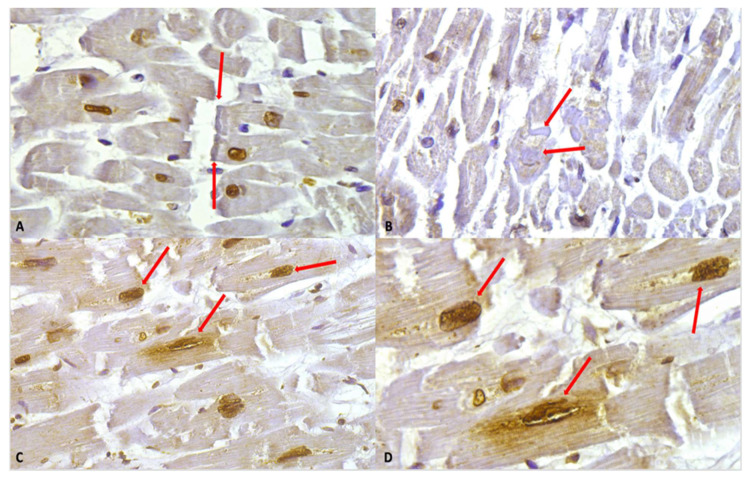
(**A**) Myofibrillar rhexis with doubling of the rupture extremity (red arrows). Cardiomyocytes nuclei immunostaining is shown, along with a slight positivity in myofibers. (**B**) Contraction bands (red arrows) in the context of myofiber moderate positivity immunostaining. (**C**) Nuclear and perinuclear positive immunostaining (red arrows) 40×. (**D**) Clearly visible at higher magnification, the nuclear and perinuclear positive (red arrows) immunostaining, 60×.

Despite the intensity of the staining, in the study group, we observed hypercontracted myocardial cells with rhexis of the myofibrillar apparatus into cross-fibre, anomalous, and irregular bands (contraction band necrosis, CBN). The necrosis was multifocal and formed by foci of a few cells; a myofibrillar rhexis with evidence of doubling of the rupture extremity, indicating that the rupture occurred at the site of a contraction band (Figure 4).

Intense immunostaining positivity in the cardiomyocytes’ nuclei was widely observed in all tested heart samples; enlarged and square-edge nuclei were observed. This “boxcar nucleus” like shape suggests cardiac myocyte hypertrophy.

Another interesting finding was related to a peri-nuclear positive immunostaining (showing slightly less intensity compared to the nuclear one).

The positive immunostaining observed in different cardiomyocyte compartments (myofibrillar or nuclear), evaluated using a semi-quantitative five-point ordinal scale (0–4), was analyzed using Spearman’s rank correlation for both cardiac samples (LLVW and IS) to assess the potential influence of the PMI on the localization of the immunohistochemical staining. All analyses yielded non-significant correlations (myofibrillar–LLVW vs. PMI: ρ = 0.180, *p* = 0.521; myofibrillar–IS vs. PMI: ρ = 0.275, *p* = 0.319; nuclear–LLVW vs. PMI: ρ = 0.277, *p* = 0.317; nuclear–IS vs. PMI: ρ = −0.117, *p* = 0.677).

The absence of significant associations between PMI and immunohistochemical expression in either myofibrillar or nuclear compartments indicates that, within the examined postmortem intervals, the detectable immunohistochemical signal in cardiomyocytes does not appear to be influenced by PMI.

## 3. Discussion

An experimental IHC model was applied to investigate postmortem anti-cocaine antibody expression and localization in heart samples from cases of cocaine-related death. The primary objective of the present study was to ascertain the reliability of IHC as a complementary or alternative diagnostic tool in the field of forensic pathology, with a view to enhancing the ability to establish drug-related death.

All examined cases demonstrated a clear and extensive positive reaction to the anti-cocaine antibody, with immunostaining exhibiting variable grades of expression and intensity within cardiomyocytes. Staining was consistently observed in both myofibrillar and nuclear compartments and showed no significant influence from the postmortem interval, at least within the range represented in our cohort (mean PMI 72.33 h, SD = 32.9; range 30–144).

In addition, positive immunostaining effectively highlighted several cardiomyocyte alterations typically associated with cocaine exposure, providing a clear visualization of structural damage. Microscopic pathological findings in cocaine-related deaths are often nonspecific and frequently insufficient for definitive forensic or judicial conclusions. Nevertheless, the histological cardiac changes linked to cocaine toxicity are of considerable diagnostic relevance. In our study, all cases displayed positive immunostaining in hypercontracted myocardial cells characterized by rhexis of the myofibrillar apparatus into cross-fiber, anomalous, and irregular bands, consistent with contraction band necrosis (CBN).

Our results may reflect the role of cocaine on cardiac homeostasis; cocaine cardiotoxicity arises from both indirect sympathetic activation and direct myocardial effects. By blocking presynaptic reuptake of catecholamines, cocaine increases dopamine and norepinephrine concentrations in the synaptic cleft, enhancing post-synaptic stimulation and sympathetic drive. Additionally, it directly impairs cardiac function through sodium and potassium channel blockade, disruption of excitation–contraction coupling, and altered calcium handling within cardiomyocytes [18,19].

Recent evidence also implicates oxidative and nitrosative stress as key contributors to cocaine-induced cardiac injury. Elevated production of reactive oxygen and nitrogen species (ROS/RNS) and mitochondrial dysfunction promote oxidative imbalance and cellular damage in the myocardium, particularly under chronic exposure [20,21,22].

Oxidative stress has been recognized as a critical contributor to cocaine-induced cardiac injury. Excessive formation of reactive oxygen species (ROS) has been implicated in numerous cardiovascular pathologies, including cardiomyopathy, ventricular remodeling, and myocardial infarction due to coronary artery vasoconstriction, atherosclerotic phenomena, and thrombus formation [23]. In the setting of cocaine exposure, ROS generation may result from activation of NADPH oxidases (NOX), secondary stimulation of xanthine oxidase, oxidative metabolism of cocaine, and catecholamine auto-oxidation induced by adrenergic overstimulation. These mechanisms converge to produce myocardial oxidative damage, promoting mitochondrial dysfunction and cellular injury that contribute to the structural and functional alterations characteristic of cocaine-associated cardiomyopathy [24,25,26].

A recent study demonstrated a significant increase in oxidative and nitrosative stress within the cardiac tissue of individuals who died after the administration of high, lethal doses of cocaine. Immunohistochemical analyses revealed enhanced expression of iNOS, NOX2, and nitrotyrosine, together with elevated levels of 8-hydroxy-2′-deoxyguanosine (8-OHdG), a marker of oxidative DNA damage. These findings indicate that excessive ROS and RNS generation contribute to myocardial injury through oxidative modification of proteins, lipids, and nucleic acids, ultimately leading to mitochondrial dysfunction and cell damage. The observed pattern supports the concept that redox imbalance and nitrosative stress represent central mechanisms in the acute cardiotoxic effects of cocaine [27,28].

The results of our immunohistochemical study may be of interest to better understand the timing of cocaine metabolism and mechanism of action: the nuclear immunostaining may reflect the cocaine-mediated oxygen DNA damage [29,30].

## 4. Materials and Methods

Fifteen cases of cocaine-related death were selected (M:F = 8:7; mean age 34.27 years). Inclusion criteria required toxicological positivity for cocaine and negativity for all other substances, including ethanol. In all cases, postmortem examination confirmed cocaine-related death. Toxicological analyses were performed on peripheral blood, urine, and bile using gas chromatography–mass spectrometry (7820A Series, Agilent, Santa Clara, CA USA).

Case characteristics and toxicological results are presented in Table 3. The mean age of the study group was 34.3 years (SD = 9.9; range 19–51), the mean BMI was 25.2 kg/m^2^ (SD = 4; range 19.8–33), and the mean PMI was 72.33 h (SD = 32.9; range 30–144). All individuals were HIV-1 negative.

The control group comprised ten cases (M:F = 6:4; mean age 43.9 years; mean BMI 84.4) who died in road traffic accidents, with no clinical history of drug abuse and negative toxicological screening for all substances. The rapid nature of these deaths meant that it was unlikely that postmortem alterations would have occurred. The controls demonstrated a uniform distribution with respect to the post-mortem interval of the study group of cocaine-related deaths.

A survey of the literature revealed a paucity of indications regarding the optimal methodology for the use of the mouse anti-cocaine monoclonal antibody in immunohistochemistry. This antibody is the only one that has been validated for use in immunohistochemistry. The antibody Ig fraction is supplied in a buffer consisting of 0.01 M phosphate-buffered saline (pH 7.2–7.4) with 0.01% NaN3 and an antibody stabilizer. The antibody was generated with an immunizing antigen-specific chemically linked carrier protein. The hybridoma was selected by ELISA-positive cloning and has been purified by affinity chromatography. The antibody is supplied by Abbo Max, San Jose, CA, USA. In the absence of analogous experimental studies, it was necessary to establish a suitable methodology and the appropriate antibody dilution from the beginning.

Samples from each case were preserved in 10% buffered formalin, then washed with phosphate-buffered saline (PBS) and dehydrated using a graded alcohol series. Following the process of dehydration, the samples were cleared in xylene and subsequently embedded in paraffin. Routine microscopic histopathological studies were performed using formalin-fixed paraffin-embedded tissue, sectioned at 4 μm, and stained with hematoxylin-eosin (H&E).

Sections were cut at 4 μm and mounted on slides for further analysis. Antigen retrieval was conducted in citrate buffer (pH 6) by microwave heating at 650 W for two consecutive 5 min cycles. Subsequently, the cocaine monoclonal antibody was applied at a 1:150 working dilution.

All statistical analyses were performed using IBM SPSS Statistics version 29 (IBM Corporation, Armonk, NY, USA), with two-tailed *p*-values < 0.05 considered statistically significant.

## 5. Conclusions

The results of this IHC study highlight an intense positive response to the anti-cocaine antibody: the positive immunostaining in cardiomyocytes was observed in all the studied cases, with clear localization in both myofibrillar and nuclear compartments. A limitation of our study is the small sample size: this factor is partially linked to precise and strict inclusion criteria when selecting the subjects. The scarcity of single cocaine-positivity cases suitable for this research may reflect the ongoing rise in polydrug consumption, including ethanol. Also, detailed immunohistochemical anti-cocaine studies are not available, which excludes a comparison of our work with others.

This study provides novel insights into the mechanisms underlying cocaine-induced cardiotoxicity [31,32]. Considering the growing evidence of oxidative/nitrosative stress and oxidative DNA damage in cocaine-induced cardiotoxicity, the observed differential postmortem immunohistochemical patterns in nuclear and myofibrillar compartments support the hypothesis that redox imbalance plays a pivotal role in the acute cardiac effects of cocaine.

## Figures and Tables

**Table 1:** Results of the semi-quantitative evaluation of the immunohistochemical findings and grading of the immunohistochemical reaction in the heart in cocaine-related deaths (in parentheses, the intracellular localization showing the highest intensity of the staining is reported).

Case	Toxicological Data (mcg/mL)			Immunohistochemical Data	
	*Peripheral Blood*	*Urine*	*Bile*	*Lateral left ventricular wall*	*Interventricular septum*
1	Coc: 0.26 BEG: 3.55	Coc: 12.43 BEG: 48.41	Coc: 3.47 BEG: 12.84	+++ (myofibrillar staining)	+++/++++ (nuclear staining)
2	Coc: 0.024 BEG: 0.003	Coc: >0.05 BEG: >0.05	Coc: -- BEG: --	++ (myofibrillar staining)	+ (nuclear staining)
3	Coc: 0.2 BEG: 1.34	Coc: pos BEG: pos	Coc: -- BEG: --	++++ (nuclear staining)	+/++ (myofibrillar staining)
4	Coc: 2.05 BEG:8.3	Coc: 36.8 BEG: 48.77	Coc: 23.81 BEG: 71.77	++++ (nuclear staining)	+++ (myofibrillar staining)
5	Coc: 2.81 BEG: 4.11	Coc: 10.99 BEG: 44.8	Coc: 7.56 BEG: 8.14	+++ (nuclear staining)	+++ (myofibrillar staining)
6	Coc: 0.134 BEG: 0.48	Coc: -- BEG: --	Coc: -- BEG: --	++ (nuclear staining)	++ (myofibrillar staining)
7	Coc: 1.352 BEG: 1.072	Coc: -- BEG: --	Coc: -- BEG: --	++ (nuclear staining)	++ (myofibrillar staining)
8	Coc: 2.80 BEG: 4.08	Coc: 10.48 BEG: 48.22	Coc: 8.41 BEG: 12	++ (nuclear staining)	++ (myofibrillar staining)
9	Coc: 1.98 BEG: 6.4	Coc: 9.98 BEG: 38.28	Coc: 8.11 BEG: 11.87	++ (nuclear staining)	++ (myofibrillar staining)
10	Coc: 2.99 BEG: 8.8	Coc: 32.86 BEG: 48.86	Coc: 25.62 BEG: 75.88	++ (nuclear staining)	++ (myofibrillar staining)
11	Coc: 2.09 BEG: 4.21	Coc: 29.62 BEG: 38.95	Coc: 23.98 BEG: 74.86	++ (nuclear staining)	++ (myofibrillar staining)
12	Coc: 1.78 BEG: 6.44	Coc: -- BEG: --	Coc: -- BEG: --	++ (nuclear staining)	++ (myofibrillar staining)
13	Coc: 2.33 BEG: 4.22	Coc: 10.02 BEG: 44.86	Coc: 8.08 BEG: 12	++ (nuclear staining)	++ (myofibrillar staining)
14	Coc: 1.98 BEG: 3.9	Coc: 10.65 BEG: 32.88	Coc: 9.89 BEG: 22.68	++ (nuclear staining)	++ (myofibrillar staining)
15	Coc: 2.86 BEG: 2.2	Coc: -- BEG: --	Coc: -- BEG: --	++ (nuclear staining)	++ (myofibrillar staining)

Coc: cocaine; BEG: benzoylecgonine.

**Table 2:** Immunohistochemical semi-quantitative scores by cardiac area examined (LLVW and IS) and by staining localization (myofibrillar and nuclear).

Case	*LLVW Nuclear*	*LLVW Myofibrillar*	*IS Nuclear*	*IS Myofibrillar*
1	1	3	3.5	3
2	1	2	1	2
3	4	1.5	4	1.5
4	4	3	4	3
5	3	3	1	3
6	2	2	2	2
7	2	2	2	3
8	2	2	3	2
9	2	1	2	2
10	4	2	4	2
11	2	2	2	2
12	3	2	3	2
13	4	2	4	2
14	3	2	3	2
15	4	2	4	2

1 = +; 1.5 = +/++; 2 = ++; 3 = +++; 3.5 = +++/++++; 4 = ++++.

**Table 3:** Case characteristics and toxicological findings.

Case	Age	Sex	BMI kg/m^2^	PMI (Hours)	Cocaine mcg/mL	BEG mcg/mL
1	30	F	21	30	0.26	3.55
2	28	F	19.8	66	0.024	0.003
3	45	F	33	89	0.2	1.34
4	48	M	28.7	144	2.05	8.3
5	51	M	22.2	93	2.81	4.11
6	33	M	27.2	90	0.134	0.48
7	21	M	20.4	119	1.352	1.072
8	19	M	26.4	35	2.80	4.08
9	33	F	23.2	44	1.98	6.4
10	24	F	25.3	80	2.99	8.8
11	45	M	28.4	77	2.09	4.21
12	41	M	20	80	1.78	6.44
13	38	F	30	64	2.33	4.22
14	30	F	27	30	1.98	3.9
15	28	M	25	44	2.86	2.2

PMI: postmortem interval. BEG: benzoylecgonine.

## Data Availability

The data that support the findings of this study are available from the corresponding author upon reasonable request.

## Abbreviations

The following abbreviations are used in this manuscript:

BEG	Benzoylecgonine
BMI	Body Mass Index
CBN	Contraction Band Necrosis
COC	Cocaine
EME	Ecgonine Methyl Ester
IHC	Immunohistochemistry
IQR	Interquartile Range
IS	Interventricular Septum
LLVW	Lateral Left Ventricular Wall
NOX	NADPH Oxidase
NOX2	NADPH Oxidase 2
PMI	Postmortem Interval
PMR	Postmortem Redistribution
RNS	Reactive Nitrogen Species
ROS	Reactive Oxygen Species
SD	Standard Deviation
8-OHdG	8-Hydroxy-2′-Deoxyguanosine
